# Implementing an electronic medication overview in Belgium

**DOI:** 10.1186/1756-0500-7-915

**Published:** 2014-12-16

**Authors:** Hannelore Storms, Kristel Marquet, Katherine Nelissen, Leen Hulshagen, Jan Lenie, Roy Remmen, Neree Claes

**Affiliations:** Faculty of Medicine and Life Sciences, Hasselt University, Campus Diepenbeek, Agoralaan Building D, B-3590 Diepenbeek, Belgium; Koninklijk Limburgs Apothekers Verbond (KLAV), Ilgatlaan 5, B-3500 Hasselt, Belgium; Faculty of Medicine and Health Sciences, University of Antwerp, Campus Drie Eiken, Universiteitsplein 1, B-2610 Wilrijk, Belgium

**Keywords:** General practice/family medicine, Quality of care, Health care organisation and management, Communication, Pharmacotherapy

## Abstract

**Background:**

An accurate medication overview is essential to reduce medication errors. Therefore, it is essential to keep the medication overview up-to-date and to exchange healthcare information between healthcare professionals and patients. Digitally shared information yields possibilities to improve communication. However, implementing a digitally shared medication overview is challenging. This articles describes the development process of a secured, electronic platform designed for exchanging medication information as executed in a pilot study in Belgium, called “Vitalink”.

**Findings:**

The goal of “Vitalink” is to improve the exchange of medication information between professionals working in healthcare and patients in order to achieve a more efficient cooperation and better quality of care. Healthcare professionals of primary and secondary health care and patients of four Belgian regions participated in the project. In each region project groups coordinated implementation and reported back to the steering committee supervising the pilot study. The electronic medication overview was developed based on consensus in the project groups. The steering committee agreed to establish secured and authorized access through the use of electronic identity documents (eID) and a secured, eHealth-platform conform prior governmental regulations regarding privacy and security of healthcare information.

**Discussion:**

A successful implementation of an electronic medication overview strongly depends on the accessibility and usability of the tool for healthcare professionals. Coordinating teams of the project groups concluded, based on their own observations and on problems reported to them, that secured and quick access to medical data needed to be pursued. According to their observations, the identification process using the eHealth platform, crucial to ensure secured data, was very time consuming. Secondly, software packages should meet the needs of their users, thus be adapted to daily activities of healthcare professionals. Moreover, software should be easy to install and run properly. The project would have benefited from a cost analysis executed by the national bodies prior to implementation.

## Findings

This article describes a pilot study called “Vitalink”, more specifically the challenges encountered during development and implementation [1]. The goal of this project is to improve the exchange of information between professionals working in healthcare and welfare in order to achieve a more efficient cooperation and better quality of care. Although aiming at a broad exchange of health and welfare information in the long run, medication management was selected as the first domain to explore potential improvement in communication amongst healthcare professionals and patients.

Sharing information about one’s health is crucial to ensure continuity and quality of care, therefore this pilot study is of high importance [2–4]. By making medical records electronically accessible, healthcare information can be consulted more easily by all healthcare professionals and by the patient. For all parties to have access to a patient’s medical record is particularly important as it improves its accuracy, which is a vital prerequisite to reduce medication errors and to enhance medication compliance [5–7]. Moreover, research shows that this reduction of medication errors through cooperation leads to a decrease in hospital (re)admissions related to medication errors [8]. Besides healthcare professionals, patients are, to some degree, capable to watch over the level of accuracy of their medication record [9, 10].

In accordance with previously published international research [6, 11], the Belgian healthcare knowledge center stresses the importance of patient involvement to establish a better communication between healthcare practitioners [12]. In the research of Blenkinsopp et al. patient involvement was also highlighted as they investigated (and confirmed) the use of paper notification cards, distributed by patients to their general practitioner and pharmacists, as a tool to improve communication [13]. However, the use of a paper tool is perceived as very time consuming. An electronic medication overview could overcome this barrier because it’s expected to be less time consuming and more accessible for healthcare professionals [13]. The use of electronic medication records is nevertheless facing other challenges such as accessibility, usability and security issues [4, 14]. To evaluate Vitalink, coordinating teams of the four project groups gathered data about these three aspects via problems reported to them by users of the electronic medication overview and through their own observations.

### Project outline

**Figure 1:**
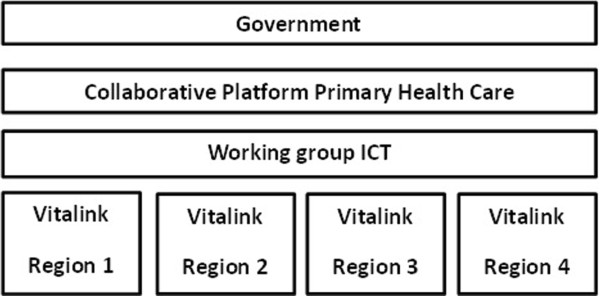
**Organization chart Vitalink.**

The pilot study of Vitalink was launched in January 2013 and ended in October 2013. This pilot study was executed in four - governmentally appointed - regions in Belgium. The development of the secured, electronic platform to exchange medication information was intertwined with the actual implementation of the digitally shared medication overview: no strict timeline was respected, decisions and changes were made prior to as well as during implementation when problems arose. To establish a broad exchange of healthcare information, healthcare professionals from across the healthcare system, as well as patient representatives, were invited to participate in the project. In each of the four regions a project group was assembled with representatives from different professional associations (general practitioners, nursing staff and pharmacists) and patient organizations. In some regions, there was also representation from the local university. Oversight of the project was confided to a steering group embed in a governmental structure, called “working group ICT”. This steering group resides under the “Collaborative Platform Primary Health Care”, an advisory board of all stakeholders in healthcare and welfare that advices the Minister of Health and Welfare and debates on priorities to be set out in health and welfare policy (Figure 1). Appointed representatives of the four project groups (“coordinating teams”) reported back to the steering committee about the progress of Vitalink in their region.

### Enrollment

Via the professional associations represented in the project group, individual healthcare professionals of primary care - general practitioners, pharmacists, nurses, home care staff - and secondary care were informed about the Vitalink project at local meetings. Given the explorative character of the project, healthcare professionals could enroll in Vitalink when interested in using the electronic medication overview, but only if the software company of their routine software had developed a software package supporting the Vitalink medication overview. In contrast, inclusion of patients who were interested in working with the electronic medication overview, was carried out by the participating healthcare professionals. Patients could only participate when having signed an informed consent. Enrollment was not subjugated to a final deadline: healthcare professionals and patients could participate from the start in January 2013 to October 2013 because implementation of Vitalink progressed even after the pilot study ended.

### Software

**Figure 2:**
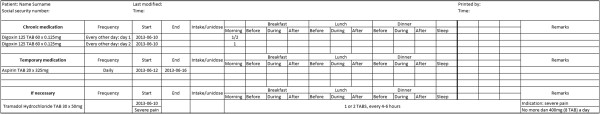
**Vitalink medication overview.**

Software companies were encouraged through financial compensation by the government to develop an electronic medication overview for patients and healthcare professionals involved in patients’ medication intake. The content and lay-out of the medication overview was discussed, modified and approved by all four project groups. The Vitalink medication overview lists all prescribed medication with name, dose, frequency, way of administration and moment of intake (Figure 2).

### Ethics

The study was approved by the Ethics Committees of the Universities of Hasselt and Antwerp. An informed consent to grant access to the medical record had to be given by the patient to make it possible for healthcare professionals to participate.

### Secured data transfer

**Figure 3:**
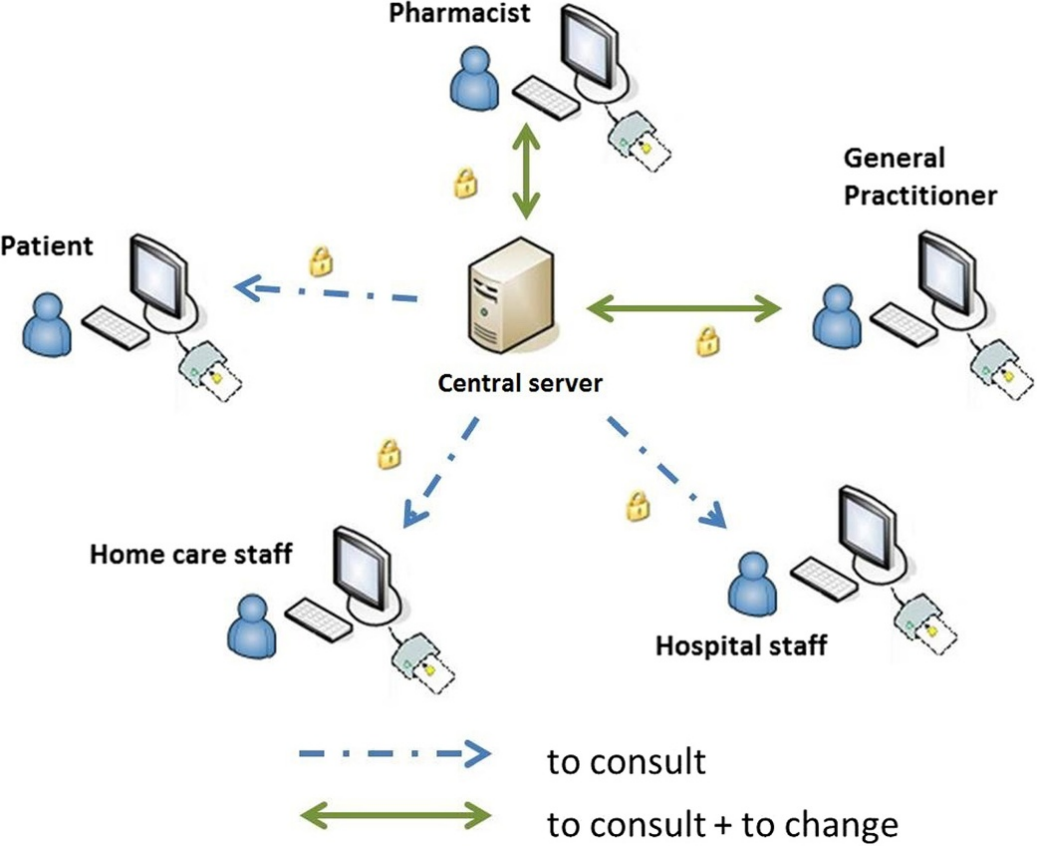
**Data transport in Vitalink.**

To exchange healthcare information, the steering committee decided, in close collaboration with the project groups, on a secured connection as described hereafter. Information itself is end to end encrypted. This means that medication information is coded both when it is centrally saved as when it is being transferred. When saving information, the Vitalink connector inside the software package encrypts the information by using a “session key”. This session key is also coded using a public encryption key. To read information two decryptors decode information. One of them being signed to a central server, designed to facilitate healthcare information exchange, called the eHealth platform, the other controlled by the “Cooperation Platform Primary Health Care”, making it impossible for each organisation to encrypt information. Only certain healthcare professionals and patients can access the healthcare information (Figure 3). Moreover, Vitalink registers every action that is performed on the healthcare information, making it possible to detect misuse of data.

### Authorized access

Project groups agreed on authorization prior to accessing healthcare information as an additional security warrantee. To have access to healthcare information the steering committee agreed on an “opt-in system”. This means that patients decide which healthcare professionals can consult their healthcare information. This informed consent can be changed by the patient at any point in time. Consent will normally be given by reading patients’ electronic identity document (eID), in the pilot study however, written consent is given on paper. The steering committee also decided to build in a second authorization check: Vitalink checks if the healthcare professional who wants to access the healthcare information has a “therapeutic relationship” with the patient. This relationship too has to be confirmed by the patient. Certain organisations, for instance those of home care staff and nurses, don’t require this confirmation. Instead, Vitalink and the eHealth platform check if the organization is authorized to access certain healthcare information.

Even if access is granted by the patient, the steering committee argued to still impose restrictions as to the actions that can be performed and the data that are available to certain healthcare professionals. General practitioners and pharmacists can create, consult and change the medication overview, whereas home care staff, nurses and secondary caregivers can only consult it. The patient can only consult the electronic medication overview. The process of consulting and changing the medication overview is described below.

### Consulting medication overview

To consult the medication overview a patient needs properly installed software, an eID, an eID reader and a connection with Vitalink over the Internet. The eHealth platform identifies the patient, sends the encrypted data to the patients’ computer, the software package decrypts the data and the patient can read the file. Healthcare professionals follow the same logging in procedure. eHealth identifies the (type of) healthcare professional and sends a Security Token Service-token (STS) valid for that session to the software package of the healthcare professional. Vitalink then checks the STS-token and retrieves information about the relationship between these healthcare professionals and the patient via the eHealth platform. Next, which data are accessible to these healthcare professionals is verified. Those data can now be consulted, after decryption. All these actions, including the identity of the healthcare professional who wants access, are registered by Vitalink.

### Changing medication overview

Only general practitioners and pharmacists were mandated to change – creating, adding, changing or deleting data – the medication overview . The same procedure as for consulting information is followed, with an additional verification whether changing the medication overview is allowed. If allowed, the changed or extra data are encrypted using a “session key”, this session key is then encrypted according to a public key. All these actions are also registered by Vitalink.

## Discussion

Coordinating teams of the four project groups gathered data about the accessibility, usability and security issues. The reflection on Vitalink is based on the experiences of the users of the electronic medication overview as reported to the coordinating teams and on the teams own observations.

### Accomplishments and limitations

Vitalink is an innovative Belgian pilot study striving for digitally exchanged healthcare information between different healthcare professionals and patients. The merit of Vitalink is to have brought together different kinds of stakeholders in healthcare in an exceptional cooperation. Despite having to deal with time constraints, insufficient resources and restrictions imposed by the government, a lot was accomplished in a short time. One of the greatest accomplishments was the development of a uniform lay-out for the medication overview. However, the implementation of the Vitalink project was subjected to various software problems. Different kinds of software packages needed to be developed, even within the same healthcare profession, causing problems to implement a uniform medication overview. Moreover, the Vitalink medication overview was not easy to consult during home visits. The requirements to have access to Vitalink, namely having an eID, an eID reader and an Internet connection, limit the accessibility of the medication overview. Vitalink software was perceived as not being adjusted to the daily practice of healthcare professionals, resulting in an extra administrative burden. Data that were already saved in healthcare professionals’ own electronic health records, were not easily transferable to the Vitalink medication overview, leading to a needless time investment. Secondly, the security check designed to identify the healthcare professional through communicating with the eHealth platform, did not always work or took a lot of time, often resulting in healthcare professionals aborting the procedure. This security procedure made it also impossible for general practitioners working in the same practice to access the Vitalink medication overview that a colleague had started.

### Reflection

As described by Terry and colleagues, when implementing new technology, it is essential to take into consideration the needs of healthcare professionals and the time investment to learn how to use the technology [14]. It seems that the security of the Vitalink system prevailed on the accessibility and consequently on the usability of the Vitalink medication overview. When information exchange between the healthcare professional’s software and the eHealth platform *interferes with the proceeding of a patient’s consultation*, the willingness to work with the Vitalink medication overview diminishes. Furthermore, because access to the medication overview requires the use of an *eID reader*, which is still not built in in most computers, the accessibility of the medication overview gets compromised, in particular during home visits. Also the need for an *Internet connection* poses difficulties when visiting patients in their homes. All these safety requirements, though important, can lead to an extra administrative burden for healthcare professionals. In turn possibly leading to *demotivated healthcare professionals* when it comes to working with this new technology. One could question whether the far driven need for data security jeopardizes the goal of this project: high quality care and safe medication use for patients.

When software is not developed according to users’ needs and when security procedures prevent actually working with the new medication overview, a lot of time is wasted [14]. Working with maladjusted software and a highly secured system in the end leads to a waste of money as well [4]. When healthcare professionals spend precious time learning how to work with new technology or spend time waiting for this technology to work, instead of being able to care for their patient, it seems that money was not spent on software that adds value to the care process. Necessary changes to adapt software in accordance to healthcare professionals’ needs will be essential to persuade healthcare professionals of the value of an electronic medication overview. These changes obviously will have its cost.

A sufficient financial investment would have been necessary to make a project like Vitalink run smoothly from the beginning [2]. Moreover, a cost analysis should have been carried out before implementing the project. This cost analysis should have included an estimation of the program and the personal cost [2]. These are the costs to develop software according to healthcare professionals’ needs, expenses for on-site training and the time investment of healthcare professionals involved in the project.

## Conclusion

A successful implementation of an electronic medication overview strongly depends on the accessibility and usability of the tool for healthcare professionals. Secured and quick access to medical data needs to be pursued. However, the identification process using the eHealth platform, crucial to ensure secured data, was very time consuming. Software packages should meet the needs of their users, thus be adapted to daily activities of healthcare professionals. Moreover, they should be easy to install and run properly. The project would have benefited from a cost analysis executed by the national bodies prior to implementation.

## Authors’ information

HS, KM, KN, NC: Hasselt University, Faculty of Medicine and Life Sciences.

LH, JL: Royal Union of Pharmacists of Limburg.

RR: University of Antwerp, Faculty of Medicine and Health Sciences.

NC: Antwerp Management School, Health Care Management.

## Abbreviations

eID: electronic identification document
ICT: Information and Communications Technology
STS: Security Token Service-token.
